# Durable Remission of Renal Cell Carcinoma in Conjuncture with Graft versus Host Disease following Allogeneic Stem Cell Transplantation and Donor Lymphocyte Infusion: Rule or Exception?

**DOI:** 10.1371/journal.pone.0085198

**Published:** 2014-01-15

**Authors:** Cornelis A. M. van Bergen, Elisabeth M. E. Verdegaal, M. Wilhelmina Honders, Conny Hoogstraten, A. Q. M. Jeanne Steijn-van Tol, Linda de Quartel, Joan de Jong, Maaike Meyering, J. H. Frederik Falkenburg, Marieke Griffioen, Susanne Osanto

**Affiliations:** 1 Department of Hematology, Leiden University Medical Center, Leiden, The Netherlands; 2 Department of Clinical Oncology, Leiden University Medical Center, Leiden, The Netherlands; Fujita Health University, School of Medicine, Japan

## Abstract

Allogeneic stem cell transplantation (alloSCT) followed by donor lymphocyte infusion (DLI) can be applied as immunotherapeutic intervention to treat malignant diseases. Here, we describe a patient with progressive metastatic clear cell renal cell carcinoma (RCC) who was treated with T cell depleted non-myeloablative alloSCT and DLI resulting in disease regression accompanied by extensive graft versus host disease (GVHD). We characterized the specificity of this immune response, and detected a dominant T cell population recognizing a novel minor histocompatibility antigen (MiHA) designated LB-FUCA2-1V. T cells specific for LB-FUCA2-1V were shown to recognize RCC cell lines, supporting a dominant role in the graft versus tumor (GVT) reaction. However, coinciding with the gradual disappearance of chronic GVHD, the anti-tumor effect declined and 3 years after alloSCT the metastases became progressive again. To re-initiate the GVT reaction, escalating doses of DLI were given, but no immune response could be induced and the patient died of progressive disease 8.5 years after alloSCT. Gene expression studies illustrated that only a minimal number of genes shared expression between RCC and professional antigen presenting cells but were not expressed by non-malignant healthy tissues, indicating that in patients suffering from RCC, GVT reactivity after alloSCT may be unavoidably linked to GVHD.

## Introduction

Allogeneic stem cell transplantation (alloSCT) is a highly effective treatment for many hematological malignancies [1]. Following HLA-matched alloSCT, the curative graft versus tumor (GVT) reactivity is mediated by donor-derived T cells recognizing minor histocompatibility antigens (MiHA) expressed by the malignant patient cells. MiHA are polymorphic peptides presented by HLA-molecules and are the result of genomic single nucleotide polymorphisms (SNP) that are disparate between patient and donor. The repertoire of patient specific MiHA can act as non-self antigens to infused donor T cells [2]. If MiHA are co-expressed by malignant cells and normal non-hematopoietic tissues, alloreactive donor T cells may induce both GVT reactivity and graft versus host disease (GVHD). Donor T cells recognizing MiHA exclusively expressed by normal and malignant hematopoietic cells from the patient can mediate GVT reactivity in the absence of GVHD. Since hematopoiesis after alloSCT is of donor origin, complete elimination of patient hematopoiesis does not impair normal hematopoiesis and immunological function. T cell depletion of the graft reduces the risk of GVHD, but increases relapse rates by abrogating therapeutic GVT reactivity. Postponed donor lymphocyte infusion (DLI) can be applied to prevent or treat disease recurrence [2], [3].

Clinical beneficial effects of alloSCT for treatment of non-hematopoietic tumors were mainly observed in patients with metastatic renal cell cancer (RCC) [4], [5] and metastatic breast cancer [6]. In RCC, alloSCT resulted in an overall response rate ranging between 20–40% [7]. In the majority of these cases, however, GVT reactivity was associated with development of clinically significant GVHD. The concurrence of GVT reactivity and GVHD indicates that tumor controlling donor T cells often recognize MiHA that are co-expressed by tumor cells and by normal tissue cells. Specific GVT reactivity and concurrent prevention of GVHD by replacement of the normal patient counterpart by donor cells, comparable to achievement of full donor chimerism in bone marrow and peripheral blood of hematological patients after alloSCT, is obviously not possible in patients with solid tumors.

For development and expansion of a primary donor-derived immune response after DLI, it may be essential that MiHA are presented by recipient-derived dendritic cells (DC) [8]. DC of patient origin can present both endogenously derived MiHA, and cross present antigens that are generated from proteins taken up from surrounding damaged tissue cells. In patients with hematological malignancies, the hematopoietic origin of DC may explain relative skewing of the T cell response towards hematopoietic cells, and targeting of hematopoiesis restricted MiHA can result in GVT reactivity in the absence of GVHD [9], [10]. Solid tumor cells and DC however, originate from different lineages and successful targeting of these malignancies may often involve MiHA that are broadly expressed not only on DC and malignant cells, but also on the normal counterpart of tumor cells.

In this study, we describe a patient with clear cell RCC who showed tumor regression and prolonged survival after alloSCT followed by DLI. Extensive chronic GVHD coincided with durable disease control but the disease became progressive when GVHD resolved. Subsequent administration of escalating doses of DLI could not re-induce the GVT reaction. We identified a strong T cell response targeting a novel MiHA (LB-FUCA2-1V) presented by HLA-B*07∶02, and induction of LB-FUCA2-1V specific T cells coincided with tumor control and GVHD. Broad recognition of GVHD target tissues by LB-FUCA2-1V specific T cells correlated with a broad expression profile of the *FUCA2* gene. Gene expression profile studies showed that, in contrast to leukemic cells, only a limited number of genes is selectively co-expressed by RCC and DC, and not by cells representing normal tissue cells. GVT reactivity may therefore be unavoidably correlated with GVHD after alloSCT and DLI for treatment of RCC.

## Materials and Methods

### Sample collection and preservation

Peripheral blood samples and skin biopsies were collected from patient, donor, and third party individuals after approval by the Leiden University Medical Center institutional review board according to the Declaration of Helsinki. Written informed consent was given by patient and donor, and by 3^rd^ party individuals to investigate materials and to publish data and case details. Peripheral blood mononuclear cells (PBMC) were isolated by Ficoll-Isopaque separation and cryopreserved. Skin biopsies were immediately processed.

### Generation and culture of cell lines

EBV-transformed B-lymphoblastic cell lines (EBV-LCL) were generated in-house from PBMC from patient, donor and third party individuals after approval by the Leiden University Medical Center institutional review board according to the Declaration of Helsinki. Written informed consent was given by patient and donor, and by 3^rd^ party individuals to investigate materials and to publish data and case details. EBV-LCL were generated and cultured in Iscove's modified Dulbecco's medium (IMDM, Lonza, Verviers, Belgium) with 10% FBS (Lonza) [11]. To obtain fibroblast and keratinocyte cell lines, single cell suspensions were generated from skin biopsies by mechanical and enzymatic dissociation. Fibroblasts were obtained by culturing in Dulbecco's modified Eagle's medium (DMEM) with low glucose (Lonza) with 10% FBS and keratinocytes by culturing in keratinocyte serum-free medium supplemented with 30 µg/ml of bovine pituitary extract and 2 ng/ml of epithelial growth factor (Invitrogen, Carlsbad, CA). RCC and melanoma cell lines were previously established in Leiden or kindly provided by Prof. A. Knuth (University of Zürich, Zürich, Switzerland) (RCC Mz1774 and RCC Mz1851) and Prof. P Straten (Danish Cancer Society, Copenhagen, Denmark, MEL SK23) and were cultured in DMEM with 8 % FBS. Immature dendritic cells were derived from monocytes isolated from PBMC using MACS CD14 MicroBead isolation (Miltenyi Biotec GmbH, Bergisch-Gladbach, Germany) and cultured for 2 days in IMDM with 10% FBS with 100 ng/ml GM-CSF (Novartis, Basel, Switzerland) and 500 IU/ml IL-4 (Schering-Plough, Bloomfield, NJ). DC were subsequently matured for 2 days by adding 10 ng/ml TNF-α (R&D Systems, Abingdon, UK), 10 ng/ml IL-1β (Immunex, Seattle, WA), 10 ng/ml IL-6 (Cellgenix, Freiburg, Germany), 1 µg/ml PGE2 (Sigma-Aldrich, Zwijndrecht, The Netherlands), and 500 IU/ml IFN-γ (BoehringerIngelheim, Ingelheim am Rhein, Germany). In selected EBV-LCL and RCC, transductions with retroviral vector pLZRS containing HLA-B*07∶02 and the truncated NGFR marker gene were performed as previously described[12].

### SNP genotyping

SNP encoding known MiHA were determined in patient and donor-derived genomic DNA extracted with Gentra Puregene Blood Kit (Qiagen, Venlo, The Netherlands). For LB-APOBEC3B-1K, LB-ARHGDIB-1R, LB-BCAT2-1R, LB-ECGF-1R, LB-MR1-1H and LRH-1, 10 ng DNA was amplified with allele specific primers using the KASPar SNP genotyping system (KBioscience, Herts, UK). For LB-EBI3-1I, LB-ERAP1-1R, LB-GEMIN4-1V, LB-MTHFD1-1Q, LB-PDCD11-1F, HwA-9 and HwA-10, 10 ng DNA was amplified in the presence of allele specific probes using Taqman SNP genotyping assays (Applied Biosystems, Foster City, CA). After amplification, fluorescent signals were analyzed on a 7900HT device running with SDS software (Applied Biosystems). Allele specific primers and probes were selected according to the manufacturer's instructions (Table S1: MiHA disparities between donor and patient).

### Cloning and testing of T cells recognizing known MiHA

Tetramers were constructed by folding peptides in biotinylated HLA-B*07∶02 monomers followed by multimerization using streptavidin conjugated to PE as previously described with minor modifications [13]. MiHA specific T cells were visualized using PE-conjugated tetramers and PE-Cy7 labeled anti-CD8 antibodies (BD Biosciences, Breda, The Netherlands). Tetramer^+^ T cells were single cell per well sorted on a FACS Aria device (BD) in 96-wells U-bottom plates (Corning, Amsterdam, The Netherlands) containing T cell medium (TCM, IMDM with 5% pooled human serum, 5% FBS and IL-2 (100 IU/ml, Chiron, Amsterdam, The Netherlands)), and stimulated with phytohemagglutinin (PHA, 0.8 µg/ml, Murex Biotec Limited, Dartford, UK) and 5×10^4^ irradiated allogeneic PBMC. Growing T cell clones were restimulated every 10 days in TCM at a concentration of 2×10^5^/ml with 1×10^6^/ml irradiated allogeneic PBMC and PHA. TCR β-chain analysis was performed using the TCRBV repertoire kit (Beckman Coulter, Mijdrecht, The Netherlands). The reactivity of T cell clones was measured after 24 h co-incubation with 3-fold stimulator cells and release of IFN-γ in culture supernatants was measured by ELISA (Sanquin, Amsterdam, Netherlands). In selected experiments, IFN-γ pretreatment (100 U/ml) of stimulator cells was performed for 24 h at 37°C and prior to co-incubation, these cells were thoroughly washed to remove IFN-γ.

### Isolation and characterization of T cells recognizing novel MiHA

Post-DLI PBMC were stimulated with irradiated (30Gy) pre-alloSCT patient derived PBMC. The next day, T cells were purified using pan T cell isolation (Miltenyi) and stained with HLA-DR-FITC (BD). HLA-DR-expressing T cells were single cell sorted and expanded as described above. Recognition of EBV-LCL was blocked with 10 µg/ml specific monoclonal antibodies for 30 min at 37°C prior to T cell addition. Whole genome association (WGAs) was performed as described previously [10], [14]. Briefly, T cell recognition of a panel of 80 EBV-LCL was mapped to a SNP genotype database containing 1.1 million SNP of each cell in the EBV-LCL test panel. The level of matching was calculated using Fisher's exact test using ‘Plink’ software [15]. For candidate gene *FUCA2* (NM_032020) sequencing, mRNA from patient and donor was isolated from EBV-LCL using Trizol (Invitrogen) and transcribed into cDNA by reverse transcriptase (Invitrogen) using oligo-dT primers (Roche Diagnostics, Almere, The Netherlands). *FUCA2* gene transcripts were amplified by PCR using forward (5′-GAATATTGGGCCCACACTAGA-3′) and reverse (5′-CATTTGCTTTCTCCATGTGC-3′) primers covering the region of interest. PCR products were analyzed by DNA sequencing, and patient and donor sequences were aligned to detect disparities. For the SNP that were identified by WGAs and gene sequencing, amino acid sequences spanning the SNP were analyzed using the online algorithm of NetMHC to search for sequences with predicted binding to HLA B*07∶02 [16]. Candidate peptides were synthesized, dissolved in DMSO, diluted in IMDM and added to donor EBV-LCL (2×10^4^/well) in 96-well U-bottom plates for 2 h at 37°C. T cells (2×10^4^/well) were added, and after overnight incubation supernatants were tested for IFN-γ production by ELISA.

### Microarray gene expression analysis

Lineage specific hematopoietic cells were purified from 3^rd^ party donor PBMC by flowcytometric sorting based on expression of CD19, CD3, and CD14. Purified malignant hematopoietic cells were obtained by flowcytometric sorting from leukemic samples for CD19^+^ cells from 2 different B-ALL patients and for CD33^+^/CD14^-^ cells from an AML-M4 and an AML-M5 patient. Non-hematopoietic normal cell lines included skin-derived fibroblasts, keratinocytes and proximal tubular epithelial cells cultured with and without IFN-γ (100 IU/ml, 2 days). Non-hematological malignant cells included renal cell carcinoma (RCC 90.03 and RCC 92.11) and melanoma (MEL SK23 and MEL 136.2). Total RNA was isolated using small and micro scale RNAqueous isolation kits (Ambion, Austin, TX, USA), and amplified using the TotalPrep RNA amplification kit (Ambion). After preparation using the whole-genome gene expression direct hybridization assay (Illumina), complementary RNA samples were dispensed onto Human HT-12 v3 Expression BeadChips (Illumina). Hybridization was performed for 17 h at 58°C and mean fluorescence intensities (MFI) were quantified using a BeadArray 500GX device. Microarray gene expression data were analysed after quantile normalization in R 2.15 (R Project).

## Results

### Clinical course

**Figure 1:**
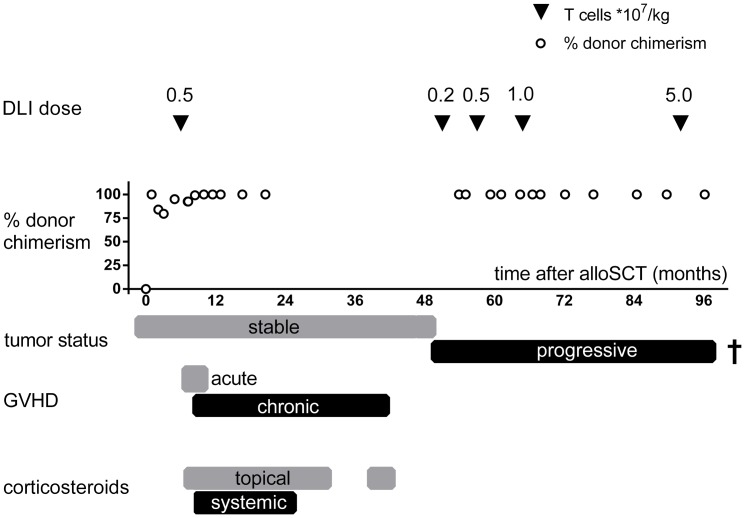
Clinical course. DLI doses, donor chimerism and the clinical course following DLI are depicted during time after allo-SCT (months, x-axis). The infused dose of T cells (filled triangles) and chimerism status (% of donor cells as measured by XY-FISH in PBMC, open circles) are shown in the upper part of the graph. Rectangles in the lower part of the graph indicate tumor status, GVHD state and GVHD treatment.

A 51 year old female patient with progressive metastatic clear cell RCC and multiple lung metastases was treated with non-myeloablative alloSCT. Prior to stem cell transplantation, the patient received a conditioning regimen consisting of Fludarabine (6×30 mg/m^2^), Busulphan (2×3.2 mg/kg), Cyclophosphamide (2×750 mg/m^2^) and horse anti-thymocyte globulin (Lymphoglobulin, 4×10 mg/kg). T cells were depleted from the peripheral blood stem cell graft derived from her HLA-identical brother by incubation with 20 mg of Alemtuzumab ‘in the bag’ [17]. Engraftment was obtained and XY-FISH analysis of PBMC showed full donor chimerism one month after alloSCT. However, incomplete donor chimerism (84%, 80%, 95% and 93%) was detected after 2, 3, 5 and 7 months, respectively (Figure 1). GVHD did not occur after alloSCT, and no change in the tumor status was observed. Seven months after alloSCT postponed DLI was administered at a single dose of 5×10^6^ T cells/kg, resulting in conversion to full donor chimerism, which persisted during the following years. Severe acute skin GVHD occurred 30 days after DLI and developed into persistent extensive chronic skin GVHD in the following years. Skin GVHD gradually resolved after prolonged topical and systemic treatment with corticosteroids (Figure 1). GVHD was accompanied with 50% reduction in size of the measurable lung metastasis and stable disease (according to RECIST criteria) for 2 years. Nearly 2 years after alloSCT a new lesion developed in the remaining kidney. The gradual resolution of chronic GVHD was accompanied by diminished GVT reactivity and growth of lung metastases 4 years after alloSCT. In an attempt to re-initiate GVT reactivity, escalating DLI doses of 2×10^6^, 5×10^6^, 1×10^7^ and 5×10^7^ T cells/kg were given at 51, 57, 64 and 92 months after alloSCT, respectively. No GVHD developed but also no GVT reactivity could be achieved and the patient died of progressive disease 8.5 years after alloSCT.

### Detection of T cells specific for known MiHA

To characterize the specificity of the immune response, we first measured SNP encoding known MiHA to detect disparities between patient and donor. Given the HLA-type of the patient, 13 MiHA were selected and analyzed by SNP genotyping assays (Table S1). The only MiHA expressed in the patient but absent in the donor, and therefore potentially allowing a donor-derived T cell response targeting patient cells, was the previously identified MiHA LRH-1, encoded by a single nucleotide deletion in the P2RX5 gene causing a frame shift. Using LRH-1 tetramers, T cells specific for LRH-1 were detected in peripheral blood at the onset of GVHD at a frequency of 0.14% of CD8^+^ T cells (data not shown). Single LRH-1 tetramer positive T cells were subsequently isolated using flowcytometry, expanded, and tested for recognition of various normal and malignant cells (data included in Figure 3A). Patient derived EBV-LCL strongly stimulated LRH-1 specific T cells, as measured by the production of IFN-γ. Recognition of patient-derived skin fibroblasts was very weak and could only be observed after pretreatment with IFN-γ. Dendritic cells (DC) and keratinocytes were not recognized. No recognition of LRH-1 positive RCC cell lines, tested either directly or after pre-incubation with IFN-γ, was observed, indicating that additional T cell responses targeting other MiHA than LRH-1 must have been involved in the immune response.

### Isolation of T cell clones recognizing the novel MiHA LB-FUCA2-1V

**Figure 2:**
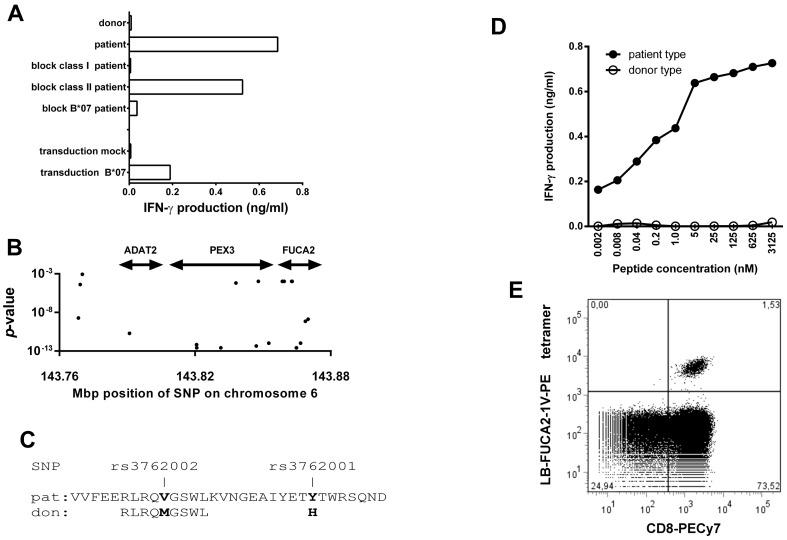
*FUCA2* encodes a novel MiHA presented by HLA-B*07∶02. (A) HLA-restricted reactivity of a T cell clone with unknown specificity was determined by testing recognition of patient EBV-LCL pre-incubated with monoclonal antibodies against HLA class-I, HLA class-II and HLA-B*07 prior to addition of T cells. In addition, mock transduced and pLZRS-NGFR-HLA-B*07∶02-transduced third party EBV-LCL were used as test cells. Reactivity was measured by Elisa and is depicted as the concentration of IFN-γ (ng/ml) in the supernatant after 24 h of co-cultivation. (B) WGAs identified a region on chromosome 6 associated with T cell recognition. Each dot represents a SNP relative to its position on chromosome 6 and the significance of association is expressed by *P* value. Double-headed arrows locate the genes *ADAT2*, *PEX3* and *FUCA2*. (C) The *FUCA2* gene contains 2 associating non-synonymous SNP. The amino acid sequence containing these SNP was investigated for potential peptide binding to HLA-B*07∶02, resulting in 1 candidate peptide sequence spanning rs3762002. (D) Synthetic peptides containing the patient specific valine residue (closed circles) and donor specific methionine residue (open circles) were loaded on donor EBV-LCL and tested for recognition by the T cell clone. (E) LB-FUCA2-1V tetramers were used to stain a patient sample collected at the onset of GVHD.

To further identify T cell responses targeting unknown MiHA in this patient, we incubated peripheral blood taken 37 days after the first DLI at the time that GVHD was apparent with pre-transplant PBMC and isolated activated T cells. Clonal expansion of CD8^+^ T cells expressing HLA-DR initially resulted in the generation of 7 MiHA-specific T cell clones (data not shown). Two T cell clones could sufficiently be expanded to allow further characterization. One T cell clone was demonstrated to be restricted to HLA-B*38∶01, as determined by using a panel of partly HLA-matched EBV-LCL (data not shown). Another T cell clone was restricted to HLA-B*07∶02 (Figure 2A), allowing characterization of the MiHA by WGAs using our panel of SNP-genotyped HLA-B*07∶02 positive EBV-LCL [10]. T cell recognition of this panel separated MiHA^pos^ and MiHA^neg^ EBV-LCL, and association between the recognition pattern and a detailed SNP genotype map of the tested EBV-LCL identified significantly associating SNPs located on chromosome 6 in a genomic region spanning three genes (Figure 2B). The majority of the associating SNP was located in non-coding regions except for rs3762001 and rs3762002, which both encoded amino acid polymorphisms in the *FUCA2* protein (Figure 2C). Predicted binding of polymorphic peptides in HLA-B*07∶02 was only found for rs3762002, which encoded a valine to methionine substitution at position 356 of the *FUCA2* protein (NP_114409). DNA sequencing of rs3762002 demonstrated the presence of the valine encoding SNP in the patient, but not in the donor (Figure 2C). Specific recognition of patient type peptide (RLRQ***V***GSWL) at nanomolar concentrations confirmed that SNP rs3762002 encoded the novel MiHA, which was designated LB-FUCA2-1V (Figure 2D). Tetramers were produced, and staining of T cells in a PBMC sample collected at the onset of acute GVHD 35 days after the first DLI, revealed 1.5% of circulating LB-FUCA2-1V specific T cells (Figure 2E). In samples taken shortly thereafter, frequencies of tetramer positive T cells strongly decreased, and became undetectable at 6 months after DLI. In line with the absence of any clinical effect following administration of escalating doses of donor lymphocytes between 4 and 8 years after alloSCT, LB-FUCA2-1V specific T cells remained undetectable. In order to detect low numbers of MiHA-specific T cells, PBMC samples were stimulated with donor-derived monocytes pulsed with LB-FUCA2-1V or LRH-1 peptide and cultured for 7 days prior to tetramer staining. LB-FUCA2-1V specific T cells were expanded to 2.64% and 0.64% of CD8^+^ T cells in samples taken 83 and 128 days after the first DLI, respectively. LRH-1 specific T cells were present at low frequencies, but could not be expanded by in vitro stimulation. Interestingly, LB-FUCA2-1V specific T cells were induced in an aliquot of the 5th DLI, illustrating that a low precursor frequency of LB-FUCA2-1V specific T-cells were present in the donor, but still remained undetectable in patient PBMC taken 90 days after this DLI (Figure S1).

**Figure 3:**
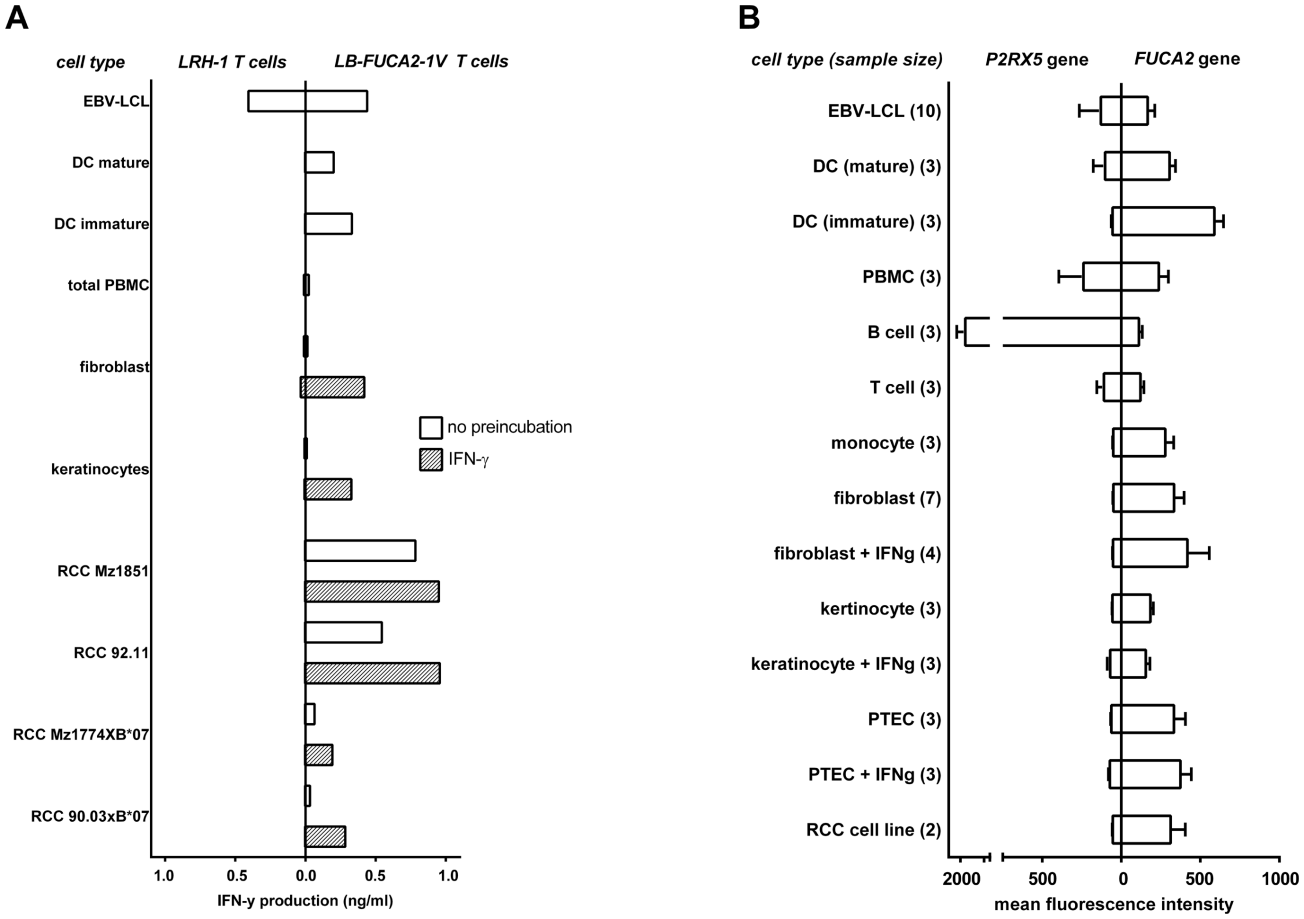
LRH-1 and LB-FUCA2-1V recognition and gene expression of *P2RX5* and *FUCA2.* (A) LB-FUCA2-1V and LRH-1 specific T cells were tested against patient-derived cells (EBV-LCL and fibroblasts) and a panel of 3^rd^ party cells expressing HLA-B*07∶02 and the LB-FUCA2-1V and/or LRH-1 MiHA. Cell lines RCC 90.03 and RCC Mz1774 were retrovirally transduced to express B*07∶02. Fibroblasts, keratinocytes and RCC cell lines were tested after 24 h pre-incubation in the absence (open bars) or presence (hatched bars) of 100 IU/ml of IFN-γ. Reactivity was measured by Elisa and is depicted as the concentration of IFN-γ (ng/ml) in the supernatant after 24 h of co-cultivation. (B) Expression patterns of the MiHA encoding genes *P2RX5* (LRH-1) and *FUCA2* (LB-FUCA2-1V) were determined by quantifying mRNA levels using microarray analysis. Expression, depicted as mean fluorescence intensity (MFI), is shown in hematopoietic cells (PBMC, B cells, T cells, monocytes, immature and mature DC and EBV-LCL), non-hematopoietic cells (fibroblasts, keratinocytes and PTEC pretreated with and without IFN-γ) and RCC cell lines. Numbers between brackets indicate the number of analyzed individual samples.

### Tissue distribution of LRH-1 and LB-FUCA2-1V

The isolated LB-FUCA2-1V specific T cells were tested for recognition of normal and malignant cells. In contrast to the isolated LRH-1 specific T cells, LB-FUCA2-1V specific T cells broadly recognized tested target cells, including mature monocyte derived DC's (monoDC) (Figure 3A). Furthermore, specific recognition of MiHA^pos^ RCC cell lines was observed, indicating a dominant role for LB-FUCA2-1V specific T cells in tumor control. Recognition of fibroblasts and keratinocytes was measured after pretreatment with IFN-γ, suggesting a role in development of GVHD. Next, we analyzed mRNA expression levels of the *P2RX5* and *FUCA2* genes, encoding the LRH-1 and LB-FUCA2-1V MiHA, respectively. The analysis confirmed B cell specific expression of the LRH-1 encoding gene *P2RX5*, in the absence of significant gene expression in other cell types. Substantial expression of the *FUCA2* gene was measured in RCC and in proximal tubular epithelial cells (PTEC), but also in fibroblasts and to a lesser extent in keratinocytes (Figure 3B). In addition, *FUCA2* mRNA was detectable in the majority of hematopoiesis-derived cells, which is in line with broad recognition of these cell types by the LB-FUCA2-1V specific T cells.

### Identification of genes specifically expressed by RCC

**Figure 4:**
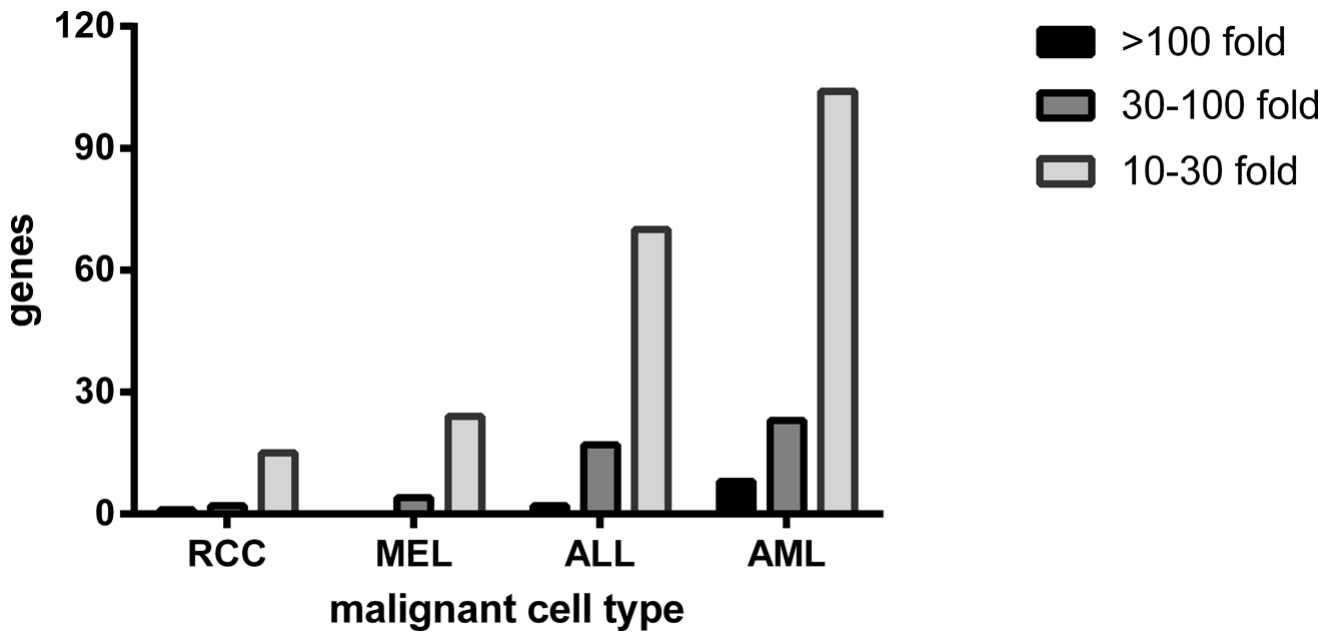
Genes over-expressed in both malignant cells and monoDC as compared to healthy tissue cells. Mature monocyte derived DC (monoDC), RCC cell lines, melanoma (MEL) cell lines, ALL and AML were investigated for gene expression using microarray techniques. For each cell type, 2 different samples were analyzed. Indicated are the numbers of genes showing more than 10, 30 or 100-fold over-expression in both the malignant cells and monoDC as compared to fibroblasts and keratinocytes. Fold over-expression was calculated from the difference in mean log-transformed values, and only genes with a significant difference (p<0.05) in mean log-transformed values as measured by a standard Student's t-test were selected.

To estimate the likelihood that a GVT reaction targeting RCC can be induced in the absence of GVHD after alloSCT and DLI, we compared gene expression profiles of RCC cell lines representing the GVT target cells, and skin-derived fibroblasts and keratinocytes representing the non-intended GVHD target cells. In addition, since the induction of effective immune responses depends on proper stimulation by professional antigen presenting cells, we also included gene expression profiles of mature monoDC. Genes with a desired expression profile were selected based on significant over-expression in both RCC cell lines and monoDC as compared to fibroblasts and keratinocytes. By setting the cutoff value for over-expression at 10-fold, 18 genes were shown to be over-expressed in RCC and monoDC (Figure 4 and Table S2). To compare these data with another non-hematopoietic malignancy, and with leukemic cells that can be targeted in the absence of GVHD, we performed similar comparisons as described above using melanoma cell lines, and ALL or AML samples instead of RCC cell lines. A similar number of genes (28 genes) was over-expressed by both melanoma cell lines and monoDC as compared to fibroblasts and keratinocytes. In contrast, the number of genes selectively over-expressed in AML and monoDC (135 genes) and ALL and monoDC (89 genes) was significantly higher (Figure 4). In conclusion, microarray gene expression analysis demonstrated that the a priori chance for beneficial GVT reactivity without GVHD in patients with solid tumors is significantly lower than in patients with hematological malignancies.

## Discussion

We analyzed T cell responses elicited after alloSCT and DLI in a patient suffering from progressive clear cell RCC. Tumor regression and stable disease was induced coinciding with severe GVHD requiring long term immune suppression which not only reduced GVHD, but also GVT reactivity. High frequencies of T cells recognizing the novel MiHA LB-FUCA2-1V were identified that strongly recognized various cell types, including RCC cells and normal tissue cells, demonstrating a dominant role for LB-FUCA2-1V specific T cells in GVT reactivity and GVHD. Broad recognition of LB-FUCA2-1V corresponded with a broad mRNA expression pattern of the encoding *FUCA2* gene. Using mRNA expression profiles, only a limited number of genes was found to be selectively expressed by RCC, but not by normal tissue cells explaining why after alloSCT and DLI for treatment of RCC, effective GVT reactivity may unavoidably be associated with GVHD.

Based on SNP genotyping for previously characterized MiHA, we first investigated LRH-1 as a potential target for a donor-derived GVT reaction. We detected low frequencies LRH-1 specific T cells in patient samples obtained after DLI, which showed selective reactivity to patient-derived EBV-LCL, but not to other cell types. This was in line with the absence of *P2RX5* transcripts encoding the LRH-1 MiHA in the non-recognized cell types. Although it has been reported that various carcinoma and melanoma cell lines may be susceptible to LRH-1 specific lysis [18], [19], we could not demonstrate recognition of MiHA^pos^ genotyped RCC cells by the isolated LRH-1 specific T cell clones. We therefore concluded that LRH-1 specific T cells may have been induced in vivo by residual patient-derived B cells at the time of the first DLI, but that the LRH-1 specific T cells were not likely to be mediators of the GVT reactivity or GVHD.

By analysing in vivo activated CD8^+^ T cells, we identified by WGAs high frequencies of HLA-B*07 restricted T cells recognizing a novel MiHA which we designated LB-FUCA2-1V. LB-FUCA2-1V specific T cells strongly recognized a variety of target cells including RCC, DC and normal skin-derived fibroblasts and keratinocytes. The broad recognition pattern, together with high levels of circulating LB-FUCA2-1V specific cells, indicated a dominant role for LB-FUCA2-1V in the GVT reaction, but also in development of severe GVHD. The *FUCA2* gene is located on chromosome 6 and encodes an enzyme called alpha-L-fucosidase that catalyzes hydrolytic cleavage of terminal fucose residues of glycoproteins [20]. Interestingly, *FUCA2* has been implicated as a factor associated with several neoplastic diseases including endometrial, oral, gastric, and hepatocellular carcinoma [21]–[24]. In vitro recognition of fibroblasts and keratinocytes by LB-FUCA2-1V specific T cells only occurred after pre-treatment with IFN-γ, whereas no difference in gene expression levels was detected. This illustrates that the immunological threshold for T cell recognition is not exclusively determined by gene expression, but also by other factors increasing the avidity of the T cell target cell interaction. After the first DLI, residual or repopulating hematopoietic cells and DC of patient origin may have initiated the T cell response resulting in an inflammatory environment. This may have up-regulated adhesion molecules like CD54 on fibroblasts rendering them susceptible to T cell induced cell lysis [25], [26]. Subsequent destruction of tissue cells amplifies the inflammation potentially resulting in the cascade leading to persistent GVHD. Re-induction of GVT reactivity by administration of escalating doses of DLI 4-8 years after the initial DLI failed. This may be due to impaired antigenic stimulation by tumor cells in the absence of patient derived APC, resulting in T cell tolerance. Furthermore, sustained signalling of T cell co-inhibitory molecules by tumor cells may have induced an exhausted T cell phenotype, resulting in a lost or reduced capacity of the tumor specific T cells to expand in vivo [27].

In hematological malignancies, alloSCT followed by postponed DLI can result in clinical remissions in the absence of GVHD [28]. We therefore explored whether GVT reactivity in solid tumors might be separable from GVHD in a similar way as graft versus leukemia can be separated from GVHD. In analogy to hematopoiesis restricted expression of MiHA that represent potentially specific targets on leukemic cells, the separation of GVT reactivity from GVHD requires targeting of MiHA that are overexpressed in RCC as compared to normal counterpart tissue cells. Effort has been made to identify such MiHA that are selectively expressed on malignant solid tumor cells, but only a few MiHA, including C19orf48 [29] and ZAPHIR [18], and a tumor associated antigen encoded by human endogenous retrovirus type E (HERV-E) [30] have been identified as targets for allo-reactive T cells in patients with RCC after treatment with alloSCT. Since it has been reported that DC are essential for the development of GVT reactivity after alloSCT, we assumed that specific GVT reactivity will occur only if the targeted genes are expressed by both DC and the malignant cell population, in the absence of expression in GVHD target tissues. Thus, the likelihood that a specific GVT reaction will occur in patients with solid tumors is determined by the number of genes that is expressed by both DC and the malignant cell population but not by normal non-hematopoietic tissues from the patient. As illustrated in figure 4, in RCC tumors only 17 genes fulfilled these criteria, whereas in AML and ALL, 133 and 92 genes respectively, were highly expressed by both DC and the malignant cells, but not in non-hematopoietic tissues, and therefore are candidate targets for a specific graft versus leukemia response. These results indicate that the likelihood of developing a tumor specific allo-immune response after alloSCT is low in patients with solid tumors, whereas in patients with hematological malignancies a significant number of targets may be identified for a leukemia specific allo-immune response. In conclusion, our results indicate that development of GVT reactivity without GVHD in patients with solid tumors is unlikely to occur, and that clinically effective T cell mediated tumor control after alloSCT and DLI in the treatment of RCC may be unavoidably linked to GVHD.

## Supporting Information

Figure S1 **Frequency of circulating MiHA specific T cells after in-vitro peptide stimulation.** Donor derived monocytes were isolated using CD14 microbeads and pulsed overnight with 0.2 µM or 1 µM of LB-FUCA2-1V or LRH-1 peptide or medium alone. Aliquots of donor lymphocytes used for DLI and samples of patient PBMC taken before and after DLI were co-cultured with peptide pulsed monocytes in the presence of 30 IU/ml IL-2. After 7 days, MiHA specific T cells were analyzed using CD8FITC and LB-FUCA2-1VPE (left panels) and LRH-1APC (right panels) tetramers, respectively. In samples containing discrete populations of tetramer^pos^ cells, the percentage of tetramer^pos^ events calculated as a percentage of CD8 T cells is depicted.
(PDF)

Table S1 **MiHA disparities between donor and patient.** The MiHA status of donor and patient was determined by performing DNA SNP genotyping using KASPar and Taqman assays.
(DOC)

Table S2 **Genes over-expressed by RCC and monoDC as compared to fibroblasts and keratinocytes.** Gene expression levels were measured on beadchip arrays and are expressed as mean fluorescence intensity. By using a cut off value of 10-fold, genes over-expressed by both RCC and monoDC as compared to fibroblasts (FB1 and FB2) and keratinocytes (KC1 and KC2) were selected.
(DOC)
