# Supplementary material for: Durable Remission of Renal Cell Carcinoma in Conjuncture with Graft versus Host Disease following Allogeneic Stem Cell Transplantation and Donor Lymphocyte Infusion: Rule or Exception?
Source: PLoS One. 2014 Jan 15;9(1):e85198. doi: 10.1371/journal.pone.0085198 (PMC3893183; doi:10.1371/journal.pone.0085198)
Supplement: Figure S1 — Frequency of circulating MiHA specific T cells after in-vitro peptide stimulation. Donor derived monocytes were isolated using CD14 microbeads and pulsed overnight with 0.2 µM or 1 µM of LB-FUCA2-1V or LRH-1 peptide or medium alone. Aliquots of donor lymphocytes used for DLI and samples of patient PBMC taken before and after DLI were co-cultured with peptide pulsed monocytes in the presence of 30 IU/ml IL-2. After 7 days, MiHA specific T cells were analyzed using CD8FITC and LB-FUCA2-1VPE (left panels) and LRH-1APC (right panels) tetramers, respectively. In samples containing discrete populations of tetramerpos cells, the percentage of tetramerpos events calculated as a percentage of CD8 T cells is depicted. (PDF) [file pone.0085198.s001.pdf]

Figure S2: Frequency of circulating MiHA specific T cells after in-vitro peptide stimulation

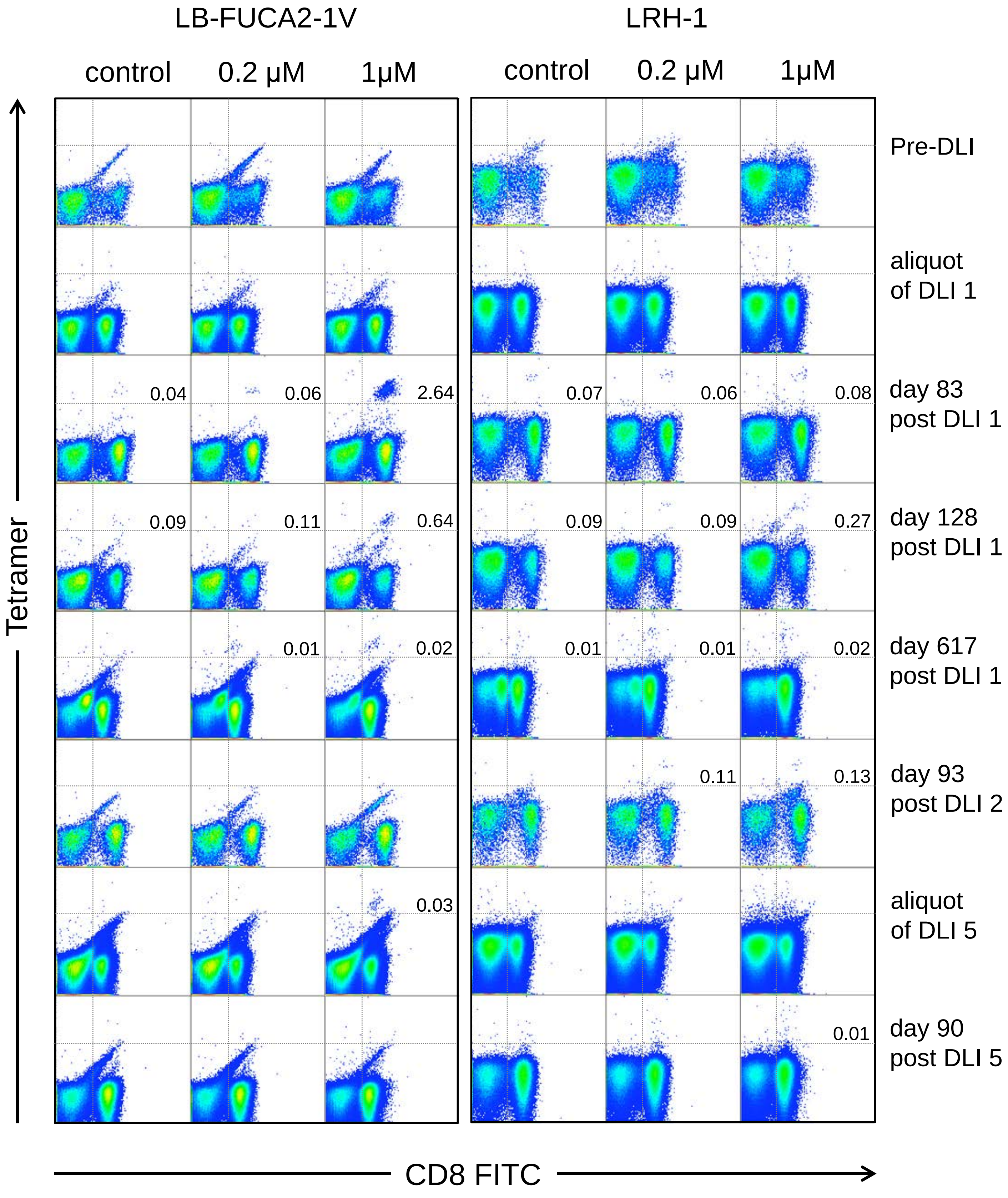

Legend to Figure S2: Frequency of circulating MiHA specific T cells after in-vitro peptide stimulation

Donor derived monocytes were isolated using CD14 microbeads and pulsed overnight with 0.2 µM or 1 µM of LB-FUCA2-1V or LRH-1 peptide or medium alone. Aliquots of donor lymphocytes used for DLI and samples of patient PBMC taken before and after DLI were co-cultured with peptide pulsed monocytes in the presence of 30 IU/ml IL-2. After 7 days, MiHA specific T cells were analyzed using $CD8^{FITC}$ and LB-FUCA2-$1V^{PE}$ (left panels) and LRH-1^{APC} (right panels) tetramers, respectively. In samples containing discrete populations of $tetramer^{pos}$ cells, the percentage of $tetramer^{pos}$ events calculated as a percentage of $CD8^{+}$ T cells is depicted.
