# Supplementary material for: Durable Remission of Renal Cell Carcinoma in Conjuncture with Graft versus Host Disease following Allogeneic Stem Cell Transplantation and Donor Lymphocyte Infusion: Rule or Exception?
Source: PLoS One. 2014 Jan 15;9(1):e85198. doi: 10.1371/journal.pone.0085198 (PMC3893183; doi:10.1371/journal.pone.0085198)
Supplement: Table S1 — MiHA disparities between donor and patient. The MiHA status of donor and patient was determined by performing DNA SNP genotyping using KASPar and Taqman assays. (DOC) [file pone.0085198.s002.doc]

**Table S1: MiHA disparities between donor and patient**

The MiHA status of donor and patient was determined by performing DNA SNP genotyping using KASPar and Taqman assays.

| MiHA | HLA restriction | Gene ID | SNP ID | SNP genomic context | Platform | Donor status | Patient status |
| --- | --- | --- | --- | --- | --- | --- | --- |
| HwA-9 | A*03 | *SP110* | rs1365776 | TCCTCTTGTACTCTCATCTTACCTC[C/T]TGGGAGGCTTTTTTTCTTATGTCTC | Taqman | pos | pos |
| HwA-10 |  | *CENPM* | rs5758511 | AGCACACCAGGCAAGTCCCACACTC[A/G]GCCCATGCACTTGCTGTTCCTCCTT | Taqman | pos | pos |
| LB-APOBEC3B-1K | B*07 | *APOBEC3B* | rs2076109 | CCTGCTCCTCTCCCAGGTGTATTTC[A/G]AGCCTCAGTACCACGCAGAAATGTG | KASPar | neg | neg |
| LB-ARHGDIB-1R |  | *ARHGDIB* | rs4703 | AGGAGGCTCCCAAGGGCATGCTGGC[C/G]CGAGGCACGTACCACAACAAGTCCT | KASPar | pos | pos |
| LB-BCAT2-1R |  | *BCAT2* | rs11548193 | GAGAATGACGAACAGGAGCGCGCGC[C/G]TGGGCTGGCTGACACCCAGCGAGGG | KASPar | neg | neg |
| LB-EBI3-1I |  | *EBI3* | rs4740 | TGTGCGGCCCCGAGCCAGGTACTAC[A/G]TCCAAGTGGCGGCTCAGGACCTCAC | Taqman | pos | pos |
| LB-ECGF-1R |  | *TYMP* | rs112723255 | GGAGGCGCTCGTACTCTCCGACCGC[A/G]CGCCATTCGCCGCCCCCTCGCCCTT | KASPar | neg | neg |
| LB-ERAP1-1R |  | *ERAP1* | rs26653 | AGCCAGCAGTGCAATTTGCTCCTGA[C/G]GGGGGTGTTCCAGGACCTGCAGGGG | Taqman | pos | pos |
| LB-GEMIN4-1V |  | *GEMIN4* | rs4968104 | AGTGGCAGATGAATTGGGACCCTGC[A/T]CTTCCACAAACCTAAGGGCAGGGAA | Taqman | pos | neg |
| LB-PDCD11-1F |  | *PDCD11* | rs2986014 | TGTTGGCCCAGATTCCTCCAAGACC[C/T]TCTTATGTCTGTCCCTCACAGGTGT | Taqman | pos | neg |
| LRH-1 |  | *P2RX5* | rs3215407 | CAGCACAGACGTTCTGCCGCTGGTT[ /G]GGGGTCACAATCAGGTTGGTGACCA | KASPar | **neg** | **pos** |
| LB-MTHFD1-1Q | DRB1*03 | *MTHFD1* | rs2236225 | GGCAATTCCTCCATCATTGCAGACC[A/G]GATCGCACTCAAGCTTGTTGGCCCA | Taqman | pos | pos |
| LB-MR1-1H | DRB3*02 | *MR1* | rs2236410 | AACCGAAATAAATTCAGGGACCCCA[C/T]GGATGGGATCCGAAACGCCCAGGCG | KASPar | neg | neg |
