# Supplementary material for: Durable Remission of Renal Cell Carcinoma in Conjuncture with Graft versus Host Disease following Allogeneic Stem Cell Transplantation and Donor Lymphocyte Infusion: Rule or Exception?
Source: PLoS One. 2014 Jan 15;9(1):e85198. doi: 10.1371/journal.pone.0085198 (PMC3893183; doi:10.1371/journal.pone.0085198)
Supplement: Table S2 — Genes over-expressed by RCC and monoDC as compared to fibroblasts and keratinocytes. Gene expression levels were measured on beadchip arrays and are expressed as mean fluorescence intensity. By using a cut off value of 10-fold, genes over-expressed by both RCC and monoDC as compared to fibroblasts (FB1 and FB2) and keratinocytes (KC1 and KC2) were selected. (DOC) [file pone.0085198.s003.doc]

**Table S3: Genes over-expressed by RCC and monoDC as compared to fibroblasts and keratinocytes**

Gene expression levels were measured on beadchip arrays and are expressed as mean fluorescence intensity. By using a cut off value of 10-fold, genes over-expressed by both RCC and monoDC as compared to fibroblasts (FB1 and FB2) and keratinocytes (KC1 and KC2) were selected.

| Gene ID | mRNA transcript ID | RCC 92.11 | RCC 90.03 | monoDC 1 | monoDC 2 | FB1 | FB2 | KC1 | KC2 |
| --- | --- | --- | --- | --- | --- | --- | --- | --- | --- |
| SPP1 | NM_000582.2 | 10535 | 14291 | 2728 | 4355 | 121 | 55 | 54 | 118 |
|  | NM_001040058.1 | 12810 | 12568 | 1140 | 6482 | 128 | 50 | 50 | 101 |
| BIRC3 | NM_001165.3 | 487 | 1020 | 14037 | 16088 | 60 | 77 | 64 | 85 |
|  | NM_182962.1 | 442 | 936 | 9879 | 18151 | 232 | 221 | 194 | 366 |
| SRGN | NM_002727.2 | 14629 | 110 | 27015 | 19122 | 179 | 46 | 111 | 255 |
|  | NM_002727.2 | 2922 | 56 | 12753 | 5399 | 64 | 52 | 54 | 142 |
| DOCK2 | NM_004946.1 | 299 | 1793 | 1502 | 2334 | 126 | 60 | 47 | 69 |
| RARRES3 | NM_004585.3 | 1095 | 774 | 16580 | 1603 | 157 | 179 | 109 | 152 |
| CD70 | NM_001252.3 | 1285 | 3336 | 641 | 165 | 49 | 54 | 90 | 52 |
| HRASLS3 | NM_007069.2 | 1584 | 845 | 9180 | 2489 | 576 | 491 | 59 | 50 |
| MARCKSL1 | NM_023009.4 | 582 | 587 | 14545 | 13561 | 167 | 148 | 182 | 419 |
| UCP2 | NM_003355.2 | 47 | 700 | 2464 | 3850 | 51 | 55 | 46 | 71 |
| BATF3 | NM_018664.1 | 281 | 406 | 3473 | 1013 | 62 | 63 | 60 | 54 |
| ITGAX | NM_000887.3 | 124 | 86 | 3260 | 2438 | 40 | 44 | 39 | 50 |
| SLC15A3 | NM_016582.1 | 564 | 809 | 13141 | 21098 | 506 | 444 | 126 | 239 |
| TBXAS1 | NM_001061.2 | 167 | 623 | 762 | 1017 | 47 | 43 | 44 | 49 |
| C15orf48 | NM_032413.2 | 264 | 121 | 4197 | 1224 | 60 | 56 | 46 | 56 |
| KYNU | NM_003937.2 | 378 | 431 | 1682 | 2196 | 44 | 45 | 70 | 314 |
|  | NM_001032998.1 | 260 | 702 | 1277 | 1434 | 49 | 49 | 66 | 193 |
| ALDH1A1 | NM_000689.3 | 55 | 2449 | 630 | 1164 | 56 | 59 | 48 | 55 |
| SEMA4D | NM_006378.2 | 85 | 378 | 1829 | 1297 | 56 | 58 | 45 | 47 |
